# Dynamic Mechanical Analysis as a Complementary Technique for Stickiness Determination in Model Whey Protein Powders

**DOI:** 10.3390/foods9091295

**Published:** 2020-09-15

**Authors:** Laura T. O’Donoghue, Md. Kamrul Haque, Sean A. Hogan, Fathima R. Laffir, James A. O’Mahony, Eoin G. Murphy

**Affiliations:** 1 Teagasc Food Research Centre, Moorepark, Fermoy, P61 C996 Co. Cork, Ireland; loddy2424@hotmail.com (L.T.O.); khaque@glanbia.ie (M.K.H.); Sean.A.Hogan@teagasc.ie (S.A.H.); 2 School of Food and Nutritional Sciences, University College Cork, T12 K8AF Cork, Ireland; sa.omahony@ucc.ie; 3 Dairy Processing Technology Centre (DPTC), Ireland; 4 Materials and Surface Science Institute (MSSI), University of Limerick, V94 T9PX Limerick, Ireland; Fathima.Laffir@ul.ie

**Keywords:** dairy powders, stickiness, thermal relaxation, spray drying

## Abstract

The α-relaxation temperatures (T_α_), derived from the storage and loss moduli using dynamic mechanical analysis (DMA), were compared to methods for stickiness and glass transition determination for a selection of model whey protein concentrate (WPC) powders with varying protein contents. Glass transition temperatures (T_g_) were determined using differential scanning calorimetry (DSC), and stickiness behavior was characterized using a fluidization technique. For the lower protein powders (WPC 20 and 35), the mechanical T_α_ determined from the storage modulus of the DMA (T_α_ onset) were in good agreement with the fluidization results, whereas for higher protein powders (WPC 50 and 65), the fluidization results compared better to the loss modulus results of the DMA (T_α_ peak). This study demonstrates that DMA has the potential to be a useful technique to complement stickiness characterization of dairy powders by providing an increased understanding of the mechanisms of stickiness.

## 1. Introduction

Stickiness of powders is a major challenge encountered by dairy processors, especially during the spray drying of products with high lactose contents, as it leads to lower powder yields and inferior powder quality. Stickiness in lactose-containing powders occurs predominantly due to the glass transition phenomenon, in which a phase change occurs in the amorphous material on exposure to high temperature and/or relative humidity (RH) conditions. This lowers the viscosity of the powder particle surface, allowing liquid bridges to form between particles, resulting in cohesion between particles and/or adhesion to equipment surfaces. A considerable amount of work has been performed developing stickiness characterization techniques that can estimate the temperature and RH conditions at which individual dairy powders will become sticky [1,2,3,4,5,6]. This information has become very useful to dairy processors at helping to minimize challenges during spray drying, allowing for the alteration of drying parameters to ensure that temperature and RH conditions within dryers are such that powder stickiness is avoided. Furthermore, these methods are also beneficial to dairy scientists to allow them to gain a deeper understanding of the wide variety of factors affecting the stickiness behavior of dairy powders.

There are a wide variety of methods available to determine the stickiness behavior of dairy powders, which can be classified as either direct or indirect techniques. Direct methods are perhaps the most accurate, as they measure the changes in a specific property of the powder, such as the viscosity or resistance to shear. One of the oldest direct stickiness measurement techniques is a propeller-driven device, first created by Lazar et al. [7] for use on tomato powder, in which the force required to move a stirrer in a bed of powder was measured. This method was later modified and used for stickiness characterization of dairy powders by Chuy and Labuza [1], Hennigs et al. [8], and Özkan et al. [3]. However, as this method is performed under static conditions, the results are likely to be more representative of the interactions that occur during storage of powders, due to the increased inter-particle surface contact [5]. In contrast, pneumatic methods, in which the particles come into direct contact with an air stream of increasing/alternating RH, may be considered more accurate, as they most closely simulate the conditions that occur during spray drying. Examples of pneumatic methods that have been used to characterize the stickiness behavior of dairy powders include the fluidization rigs used by Hogan et al. [5] and Murti et al. [9], the blow test method developed by Brooks [10] and Paterson et al. [2], and the particle gun created by Zuo et al. [4]. However, the stickiness data generated from these methods can also differ due to differences in air velocities [9], particle trajectories, and contact times between particles and the air stream.

One indirect approach that is well established and commonly used as an indication of stickiness in dairy powders is the determination of glass transition temperature (T_g_). The T_g_ can be defined as the temperature at which the glass transition takes place and is normally determined either by measurement or estimation using mathematical modeling, such as the Couchman–Karasz equation [11]. The measurement approach is considered more precise, as it allows tracking of changes that occur in a specific property of the material during the phase change. For example, differential scanning calorimetry (DSC) measures the changes that occur in specific heat capacity of the sample during the glass transition and has been widely used to determine the T_g_ of dairy powders [1,6,12,13,14,15,16,17,18]. It should be noted that while the glass transition determination is not a stickiness test method, a relationship does exist between the T_g_ and sticking point temperature (SPT), which can be used to roughly estimate the sticking temperature. One of the first studies to compare the T_g_ to the SPT, which was determined using the method by Lazar et al. [7], reported that the SPT was approximately 10–15 °C higher than the T_g_ onset [19]. Furthermore, the extent to which the temperature must exceed the T_g_ in order for sticking to occur is not consistent, even for the same powder, as it depends on a wide variety of factors, such as the powder composition [5], exposure time [20], and the methods used to determine both the SPT and T_g_ [21]. This is evident in the range of T–T_g_ values that have been reported for skim milk powder (SMP); 20.6 °C [18], 29 °C [5], 33.6 °C [22], 14–22 °C [13], and 23.3 °C [8]. The determination of T_g_ alone is therefore not an accurate method for stickiness characterization, as although there is a correlation between the SPT and T_g_, it is difficult to predict the precise temperature above the T_g_ that sticking will occur [21]. Hence, further research is required in order to develop an empirical relationship for predicting SPT, using T_g_.

Another indirect method that has recently been related to the stickiness phenomenon is dynamic mechanical analysis (DMA), also referred to as dynamic mechanical thermal analysis (DMTA), which has been used in many studies to determine mechanical α-relaxations of amorphous food materials [6,23,24,25]. Mechanical α-relaxations describe the changes in the physical state of the material around the glass transition and could therefore also be good indications of the changes in viscosity that occur during stickiness development. Furthermore, as DMA is a highly sensitive method, it may provide an opportunity to develop a greater understanding of the mechanisms of stickiness development (i.e., changes in viscoelastic behavior) when the results are compared to other binary (i.e., sticky/non-sticky) methods. The DMA method involves subjecting the sample to a sinusoidal force and measuring the amount of energy stored (storage modulus) and lost (loss modulus) from the sample. During the glass transition, as the material “relaxes” from an amorphous into a crystalline state, there will be a sudden decrease in the storage modulus (E′) and a corresponding increase in the loss modulus (E″). Silalai and Roos [6] compared the results obtained from DMA to a sticky point tester, modified from the design by Lazar et al. [7], and found that the α-relaxation temperatures (calculated from the peak of the loss modulus) were good indicators for stickiness development for SMP/maltodextrin mixes. However, the sticky point tester used in that study is a viscometry-based technique, which may not produce the most accurate stickiness characterization results compared to pneumatic methods. Furthermore, the SMP/maltodextrin powders examined in that study are also not representative of the wide range of dairy powders available commercially. For example, the primary carbohydrate in the majority of the powders was maltodextrin (rather than lactose), and the highest protein content powder examined in that study was that of the original SMP (~35% *w*/*w*).

The current study compared the α-relaxation temperatures (T_α_) derived from DMA storage and loss moduli to methods more commonly used to measure phase transitions (DSC) and stickiness behavior (fluidization) for a selection of whey protein concentrate (WPC) powders. While this provided useful information relating to the effect of measurement technique on thermally induced phase changes, the primary objective of the study was not to make a simple comparison of techniques but rather to interpret the data as a whole for the purpose of better understanding the mechanism of stickiness. In particular, it was expected that the increased understanding of the viscous and elastic transitions obtained using DMA would complement DSC and fluidization analyses to enhance the suite of tools available for the development of powdered dairy products. 

## 2. Materials and Methods

### 2.1. Materials

Whey protein concentrate (WPC) 80 powder and whey permeate powder (WPP) were supplied by local dairy companies. Model WPC powders were produced for this purpose of this study by mixing the WPC 80 and WPP and reconstituting with water in different proportions to produce WPCs with target protein contents of 20%, 35%, 50%, and 65% (*w*/*w*). These WPC80/permeate solutions were then spray dried using an Anhydro three-stage drier with fines return to the top of the drier (SPX Flow Technology, Soeborg, Denmark), using a two-fluid nozzle atomizer. Solid contents of the concentrates were 42%, 40%, 36%, and 32% for the WPC 20, 35, 50, and 65, respectively. All powders were dried using inlet and outlet temperatures of 180 °C and 80 °C, respectively, and the final stage of drying was completed in an external fluid bed at 60 °C. The WPC powders were then stored in foil bags at 16 °C prior to analysis. All analysis was carried out within six months of manufacture.

### 2.2. Powder Composition

Protein content was determined using a LECO Nitrogen Analyser FP-638 (LECO Corporation, St. Jospeh, MI, USA), using a nitrogen-to-protein conversion factor of 6.38. Fat content was determined by Röse–Gottlieb [26]. Ash content was analyzed after overnight incineration in a muffle furnace at 550 °C. Free moisture was determined after drying in an oven at 86 °C for 6 h. Lactose content was calculated by difference. Particle size was measured by laser light scattering using a Mastersizer 3000 (Malvern Instruments Ltd., Malvern, UK, equipped with an Aero S dry powder dispersion unit. The optical parameters used were refractive indices of 1.46 and 1 for powder and air, respectively; absorbance index of 0.1. Volume mean diameter D4,3 was used to characterize the size of particles.

### 2.3. Surface Composition

X-ray photoelectron spectroscopy (XPS) was carried out using a Kratos AXIS Ultra spectrometer (Kratos Analytical Ltd., Manchester, UK). The percentage coverage of protein, fat, and lactose at the powder surface was calculated according to the method described by Faldt et al. [27] in which a matrix formula is created from the elemental compositions of the various milk components. Analysis was carried out in duplicate.

### 2.4. Moisture Sorption Isotherms

Moisture sorption isotherms were determined using a dynamic vapour sorption (DVS) Intrinsic 1 (Surface Measurement Systems Ltd., London, UK). Samples (~35 mg) were first equilibrated to 0% RH and then humidified up to 90% RH in 10% increments at 25 °C using a single ramp method. Equilibrium was considered to be reached when the % change in mass with time (dm/dt) was <0.0033%/min for at least 10 min at each RH.

### 2.5. Stickiness by Fluidization

In the current study, a fluidization technique, previously described by Hogan et al. [5], was used to determine the SPT (T_f_) of each powder. Stickiness curves were generated for each sample by plotting the air (dry bulb) temperature against the RH (calculated from the saturated air temperature and absolute humidity) at which fluidization ceased.

### 2.6. Powder Equilibration

Powder samples (2 g) were transferred into glass vials and dried overnight in a vacuum oven (Jeio Tech 665 L Vacuum Oven OV-12, Fisher Scientific, Leicestershire, UK) at 45 °C. The dried samples were equilibrated in evacuated desiccators over saturated salt solutions of LiCl, CH_3_COOK, MgCl_2_, and K_2_CO_3_ (Sigma Chemical Co., St. Louis, MO, USA), with corresponding relative water vapor pressures (RVPs) of 11.4%, 23.1%, 33.2%, and 44.1%, respectively, at room temperature (23–24 °C) for 14 days.

### 2.7. Differential Scanning Calorimetry

A differential scanning calorimeter (DSC Q2000; TA Instruments, Crawley, UK) was used to determine the glass transition temperatures (T_g_) of the equilibrated powders, as described by Murphy et al. [28]. Hermetically sealed DSC aluminum pans, containing ~16 mg of powder, were heated in a nitrogen purged environment using an empty aluminum pan as a reference. The samples were subjected to the following thermal profile; heating from approximately 40 °C below to 40 °C above the T_g_ at 5 °C min^−1^, cooling back to 50 °C below the T_g_ at 10 °C min^−1^, and finally heating at 5 °C min^−1^ to an end temperature of 50 °C above the T_g_. The T_g_ onset values were determined from the second heating cycle using the TA Universal Analysis software. All analyses were completed in duplicate. T–T_g_ values were calculated by extracting the equation of the lines for the stickiness and glass transition curves and subtracting the *y* values at a given RH (*x* value).

### 2.8. Dynamic Mechanical Analysis

A dynamic mechanical analyzer (DMA Q800, TA Instruments, New Castle, UK) with 35 mm dual cantilever clamp was used to determine the α-relaxation temperatures (T_α_) of the equilibrated powders. Approximately 400 mg of equilibrated powder was loaded into a stainless-steel powder sample tray and the surface of the powder bed was leveled off and covered with a stainless-steel lid. The powder sample tray and lid were then inserted into the clamp and tightened using a screwdriver with a set torque (level 8). The analyses were carried out dynamically at a heating rate of 2 °C/min, from approximately 50 °C below the onset temperature of the decrease in storage modulus to 50 °C above the onset temperature at frequencies of 1.0, 5.0, 10.0, and 20.0 Hz. However, it was found that there was no significant difference in the temperature at which the storage modulus decreased at frequencies greater than 10.0 Hz. Therefore, all the T_α_ values were determined at 10 Hz. T_α_ was determined from the onset in the decrease in the storage modulus (T_α_ onset), and the peak of the loss modulus (T_α_ peak), using the TA Universal Analysis software (TA Instruments, New Castle, UK). A liquid nitrogen tank (50 L; CFL-50, Cryofab Inc, Kenilworth, NJ, USA) was connected to the dynamic mechanical analyzer for cooling below room temperature. The T_α_ of each powder with various RVPs was measured in duplicate. Prior to sample analysis, the dynamic mechanical analyzer was regularly calibrated using a stainless-steel bar.

### 2.9. Statistical Analysis

The results presented are the average of at least three measurements and are reported as mean value ± standard deviation. Stickiness by fluidization was performed in quadruplicate. Powder particle size was performed in triplicate. Other measurements were performed in duplicate. Statistical analysis was carried out by subjecting data sets to one-way ANOVA with a Fisher post-hoc test using Minitab 17 (Minitab LLC, State College, PA, USA) statistical analysis package. A level of confidence of *p* < 0.05 was used.

## 3. Results and Discussion

### 3.1. Bulk and Surface Composition

The bulk composition and particle size of each powder is reported in Table 1. Protein contents for the WPC 20, 35, 50, and 65 powders were 19.3, 35.7, 53.4, and 69.1 (% *w*/*w*), respectively. Lactose contents ranged from 66.2–17.5 (% *w*/*w*) for the WPC 20 and WPC 65, respectively. Fat content increased (1.31–5.33% *w*/*w*) and ash content decreased (7.34–4.23% *w*/*w*) with increasing protein content. Particle size has also been shown to affect the stickiness behavior of dairy powders [18]; however, in the present study, there was very little difference in particle size between the four powders (D4,3 values of 106–118 µm), therefore it is unlikely to be a contributing factor in their stickiness behavior.

Protein coverage at the particle surface increased with increasing bulk protein content (46.8% to 59.2% for the WPC 20 to 65), with a corresponding decrease in lactose coverage (Table 2). As expected, fat was over-represented at the particle surface compared to the bulk of the powder [29,30,31,32], and surface fat coverage increased with increasing bulk fat content [29,30].

### 3.2. Moisture Sorption Isotherms

**Figure 1:**
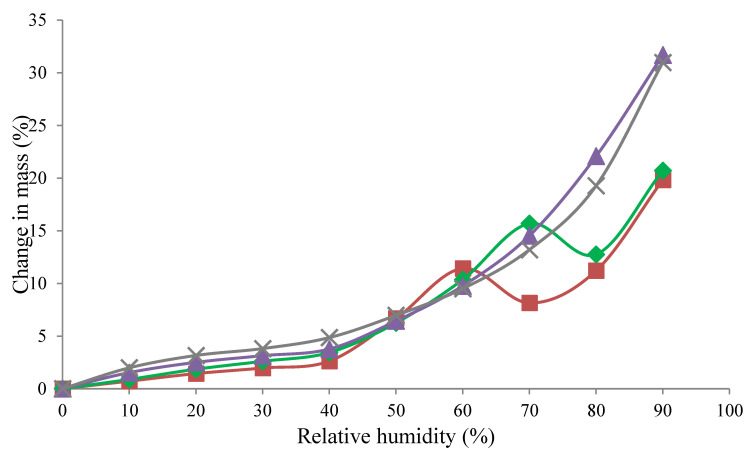
Change in mass (%) of whey protein concentrate (WPC) powders during sorption from 0 to 90% relative humidity (RH) at 25 °C; (■) WPC 20, (♦) WPC 35, (▲) WPC 50, and (X) WPC 65.

Moisture sorption isotherms for the WPC powders, ranging from 0 to 90% RH, are presented in Figure 1. The two higher protein powders showed larger final increases in mass (31.0% and 31.7% increase for the WPC 65 and 50, respectively) compared to the lower protein powders (20.7% and 19.8% increase for the WPC 35 and 20, respectively). Shrestha et al. [30] and Maidannyk et al. [33] reported similar results for a range of SMP/lactose mixtures and milk protein concentrate (MPC) powders, respectively. At RH < 40%, moisture absorption was primarily determined by the protein content of the powder, with the WPC 65 absorbing the most moisture. Lactose crystallization is evident in the isotherms for the WPC 20 and 35 by a decrease in the mass of the powders, which occurs due to the release of moisture during crystallization [12,15,33,34,35,36]. Lactose crystallization also occurred at a higher RH for the WPC 35 (~70% RH) compared to WPC 20 (~60% RH), due to the competitive/preferential sorption of water by proteins, delaying the onset of crystallization [12,14,30,34]. For the WPC 50 and 65 powders, no crystallization was evident from the isotherms, presumably due to the high protein content hindering the movement of the lactose molecules and/or the moisture released during crystallization being re-absorbed by protein [33,36].

### 3.3. Glass Transition Temperature Determination

The T_g_ onset of the WPC powders are reported in Table 3. As expected, all four powders showed a decrease in T_g_ onset with increasing water activity (a_w_) [12,13,14,16,30,33]. This is due to the plasticizing effect of water on the amorphous material, which increases the molecular mobility of the system, resulting in a decrease in T_g_ [19]. Studies have shown that the amorphous lactose content is the main determinant of the T_g_ in dairy powders [16,30]. In the current study, the T_g_ onset was also found to decrease with increasing lactose content. This trend was more pronounced in samples with a_w_ ≥ 0.33. This may be due to the increased moisture availability in higher a_w_ samples, resulting in increased water plasticization of the amorphous lactose.

### 3.4. Powder Fluidization Analysis

**Figure 2:**
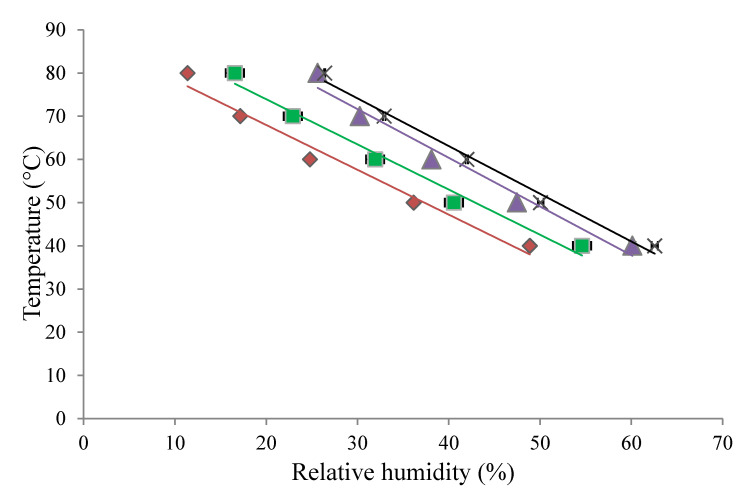
Stickiness curves for whey protein concentrate (WPC) powders; (♦) WPC 20, (■) WPC 35, (▲) WPC 50, and (X) WPC 65, determined using the fluidization technique.

Stickiness curves for each powder were generated using the fluidization approach by plotting the dry bulb temperature against the RH at which sticking occurred (Figure 2). The area above the stickiness curve represents the temperature and RH conditions where problems with stickiness are likely to occur, whereas the area below the curve represents the conditions considered safe during spray drying. For all four powders examined, as the dry bulb temperature increased, the RH at which the powder became sticky decreased. The susceptibility of the powders to sticking decreased in the order WPC 20 > WPC 35 > WPC 50 > WPC 65, with WPC 65 demonstrating the least sticky behavior. This was expected, as the stickiness of dairy powders has been shown to decrease with increasing protein content [16,36]. There is limited information available on the stickiness characterization of WPC powders; however, the SPT results obtained for the WPC 35 powder are similar to those reported by O’Donoghue et al. [18] for SMP using the same fluidization method.

### 3.5. Dynamic Mechanical Analysis

**Figure 3:**
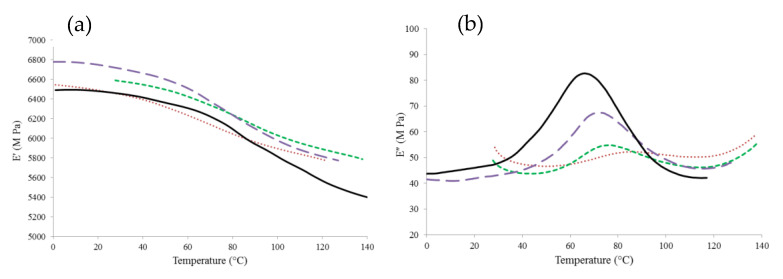
Storage (**a**) and loss (**b**) moduli of whey protein concentrate (WPC) 65 powder at selected water activities (a_w_) of 0.11 (- -), 0.23 (**‒ ‒**), 0.33 (**― ―**), and 0.44 (**――**).

Figure 3 shows the mechanical α-relaxations for the WPC 65 over a range of a_w_ (0.11–0.44). As expected, significant changes occurred in the molecular mobility of the powder with increasing temperature. The magnitude of these changes, especially for the loss moduli (Figure 3b), were found to increase with increasing a_w_, and this general trend was evident in all powders examined. The increased magnitude of the changes with increasing a_w_ is a result of the plasticizing effect of water, which increases the molecular mobility of the system [6]. This causes a decrease in the viscosity of the particle surface, leading to the onset of sticking [37].

**Figure 4:**
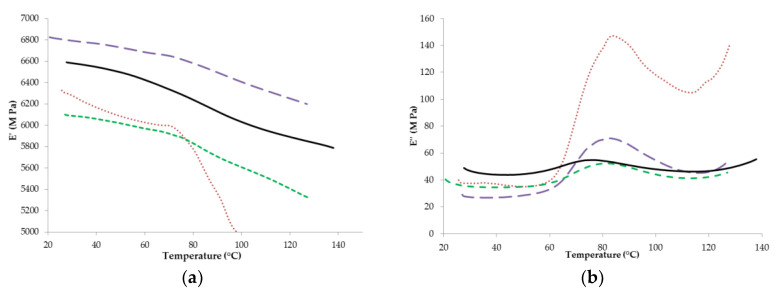
Storage (**a**) and loss (**b**) moduli of various whey protein concentrate (WPC) powders; WPC 20 (- -), WPC 35 (**‒ ‒**), WPC 50 (**― ―**), and WPC 65 (**――**), at a water activity (a_w_) of 0.23.

In the current study, the magnitude of the changes in the α-relaxations was also found to be dependent on powder composition (Figure 4). The higher the protein content of the powder, the smaller the magnitude of the changes in the moduli (Figure 4). Many studies [6,17,24,33] also observed that increasing the protein content of dairy systems led to smaller temperature induced changes in the magnitude of the moduli, when measured using DMA. This suggests an increase in the stiffness of these samples, which is likely due to the higher molecular weight of proteins, compared to lactose. However, for the present study, similar to the effect of a_w_, this trend was more pronounced in the loss moduli compared to the storage moduli. As the storage modulus is a measure of the elasticity/stiffness of a material [38], it is likely that changes in the stiffness of the sample are more subtle compared to the loss modulus, which indicates changes in viscosity.

**Figure 5:**
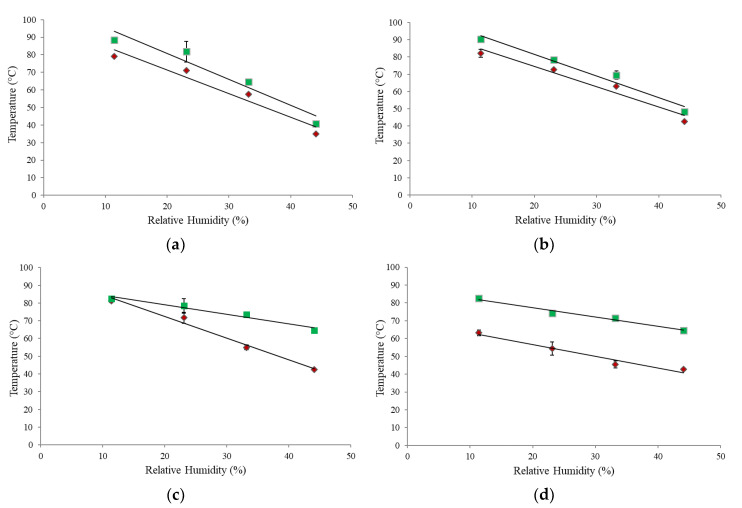
α-relaxation profiles determined from the T_a_ onset (♦) and the T_a_ peak (■) values of the dynamic mechanical analysis (DMA) method for whey protein concentrate (WPC) powders; (**a**) WPC 20, (**b**) WPC 35, (**c**) WPC 50, and (**d**) WPC 65.

The DMA profiles or “curves,” generated from the α-relaxation temperatures of the storage and loss moduli, are presented in Figure 5. The α-relaxation temperatures used to generate the stickiness curves were (a) T_α_ onset (determined from the onset of the decrease in the storage modulus) and (b) T_α_ peak (determined from the peak of the loss modulus). All analysis was carried out at the same frequency (10 Hz), as the α-relaxation temperature has been shown to be frequency dependent [6,23,39]. Figure 5 shows that T_α_ onset values were consistently lower than T_α_ peak values for all powders, as expected [39,40]. For the WPC 20 and 35 powders, the T_α_ onset and T_α_ peak results were in good agreement, with average delta T (∆T) values across the four water activities of 8.26 ± 2.27 °C and 6.42 ± 1.29 °C for WPC 20 and 35, respectively. The T_α_ peak data obtained for the WPC 35 also compare well to T_α_ peak values (i.e., X⁰ vs. Y⁰ at Z a_w_) reported by Silalai and Roos [23] for SMP at the same frequency (10 Hz). The average ∆T between the T_α_ onset and T_α_ peak values for the WPC 50 was slightly greater at 12.2 ± 9.85 °C; however, ∆T at high a_w_ was much more pronounced (~20 °C), as can be seen in Figure 5c. For WPC 65, the average ∆T was the greatest of all the powders at 21.8 ± 3.09 °C. Studies comparing the α-relaxation temperatures determined from the storage and loss moduli measured using DMA methodology reported a difference of ~20 °C [39] and ~17 °C [40] between the T_α_ onset and T_α_ peak values for samples of amylopectin and spaghetti, respectively.

Similar to the stickiness results obtained from the fluidization method, T_α_ onset was found to decrease with increasing a_w_ for both moduli (Figure 5). Silalai and Roos [23] and Maidannyk and Roos [17] also observed a similar effect of a_w_ on T_α_ peak for selected dairy powders using DMA. However, unlike the fluidization results, there was no clear influence of protein/lactose content on the T_α_ values of the WPC powders from the results determined from either modulus across the range of water activities. In contrast, other studies [17,23,24,33] have generally found that the presence of protein increased the T_α_ peak values of dairy powders.

### 3.6. Comparison of α-Relaxation, Stickiness, and Glass Transition Curves

**Figure 6:**
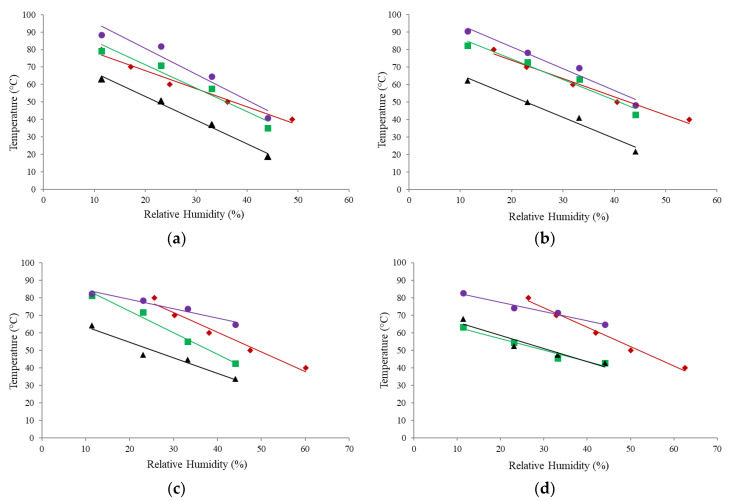
Stickiness curves for the fluidization technique (♦) and α-relaxation profiles for the storage (■) and loss (●) modulus of the dynamic mechanical analysis (DMA) method, and the glass transition curve (▲) for the whey protein concentrate (WPC) powders; (**a**) WPC 20, (**b**) WPC 35, (**c**) WPC 50, and (**d**) WPC 65.

The T_α_ values determined from the storage and loss moduli of the DMA method were compared to the stickiness curves (obtained using the fluidization method) and the glass transition curves (Figure 6). For the lower protein powders (WPC 20 and 35), the T_α_ onset results were closer to those generated using the fluidization method, compared to the T_α_ peak results. Furthermore, for these powders, the stickiness curves generated using the fluidization method and the storage moduli of DMA were almost identical (Figure 6a,b). In contrast, for the higher protein powders (WPC 50 and 65), the T_α_ peak results were closer to the fluidization results. Figure 6 also demonstrates that as the protein content of the powder increased (i.e., lactose content decreased), the T_α_ onset curve moved away from the fluidization curve and closer to the glass transition curve. Furthermore, for the WPC 65 powder, the T_α_ onset results of DMA and the glass transition curve were almost indistinguishable. This would suggest that, for powders with higher protein contents, the T_α_ onset values obtained from the DMA method may be more representative of the changes occurring during the glass transition rather than stickiness development.

It has been reported that the mechanical α-relaxation behavior, measured using DMA, follows the mobility of the lactose in the milk protein matrix [6]. For the current study, considering that protein and lactose exist in separate phases in dairy solid systems, it is likely that the higher protein content of the WPC 65 retarded the movement of the lactose, consequently affecting the structural relaxations. Fan and Roos [24] found a similar effect of protein on the enthalpy relaxations measured by DSC in lactose/protein mixes. The authors concluded that the presence of protein could affect the enthalpy relaxation results by physically blocking the movement of the lactose. It may therefore be the case that, for samples with higher protein contents, the stiffness of the sample is so great that the storage modulus determined using DMA and DSC are measuring the same structural relaxation changes. Furthermore, it should also be noted that the DMA method has been frequently used for determination of glass transition [38,39,40,41,42,43,44]. However, there is little consensus in the literature as to which DMA variable relates to T_g_, i.e., some publications report the drop of the storage modulus as T_g_, whereas others use the peak of the loss modulus or the onset/peak of the tan curve [38].

As previously mentioned, in a study by Silalai and Roos [6] the authors compared the results from DMA to the stickiness method modified from the design of Lazar et al. [7] and concluded that the DMA method was a good indication of stickiness in SMP/maltodextrin mixtures. However, the method developed by Lazar et al. [7] is a propeller-driven viscometry technique and, like the DMA method, is performed under relatively static conditions. In contrast, the fluidization rig-based approach used in the current study is a pneumatic technique performed under dynamic conditions. These two types of methods (static vs. dynamic) therefore measure particle interactions under very different conditions. First, static techniques often involve the humidification of powders in desiccators until a desired water activity is reached, which may take days, or even weeks, to complete. Furthermore, the viscometer-based technique requires an additional 20–30 min of pre-conditioning before testing in order for the sample to equilibrate to the desired temperature [16]. This may lead to physical changes within certain components of the powder, especially at higher water activities, e.g., water migration and lactose crystallization. In contrast, the powder in the fluidization apparatus undergoes a very short conditioning time of several seconds, as the powder comes in contact with the fluidizing air; the particle interactions for both methods are also very different. The powder in the viscometer-based technique is in the form of a bed, where particle interactions would be high due to the close contact. However, in the fluidization method, the particles are suspended in a stream of air and would therefore come into contact less frequently, compared to the viscometer technique. Therefore, it is quite likely that these two methodological approaches would produce different stickiness results. An example of this can be seen in the study by Murti et al. [9] in which the authors reported a 10–15 °C difference in the SPTs of the same powder when measured using a fluid bed and a particle gun. Although these are both pneumatic methods, the air velocities and particle trajectories vary greatly between the two methods. Similarly, in the current study, the SPT/T_α_ onset reported for the WPC 65 powder at an a_w_ of approximately 0.33 were very different at 70 °C and 45 °C, for the fluidization and DMA method, respectively.

### 3.7. Comparison of T–T_g_ Results from Different Measurement Techniques

As previously mentioned, the temperature at which sticking occurred in dried amorphous carbohydrate solutions was reported to be approximately 10–15 °C above the T_g_ [19]. The T–T_g_ therefore represents the temperature increment above the T_g_ at which the decrease in surface viscosity is sufficient in order for sticking to occur. Many studies have demonstrated that the T–T_g_ for dairy powders depends on factors such as the powder composition [5] and measurement techniques used [21]. The current study therefore provides an opportunity to compare the various T–T_g_ values obtained for the same powders using different stickiness measurement techniques.

The T–T_g_ values for the fluidization and DMA method (T_α_ onset and T_α_ peak) at selected points along the stickiness curves are provided in Table 4. The T–T_g_ values were determined at two points along the curves for comparison; first, at the midpoint (x value) of the stickiness curves, and second, at 15% RH. This RH was chosen as it was considered representative of industrial spray drying conditions [45]. T_f_–T_g_ results for the DSC and fluidization method, using the midpoint of the stickiness curves, ranged from 18.0–23.2 °C across the four water activities, but did not display any obvious trends. In contrast, the T_f_–T_g_ values determined at 15% RH show a general trend of increasing T–T_g_ with increasing protein content, with the exception of the WPC 65 (Table 4). In a study by Hogan and O’Callaghan [36], the authors reported that T–T_g_ (determined from the midpoint of the stickiness curves) increased with increasing protein content for selected dairy powders. This is likely due to the preferential sorption of water by the proteins, which delays the rate at which the glass transition occurs, therefore delaying the development of stickiness [36]. However, it should also be noted that, in the study by Hogan and O’Callaghan [36], the authors used the Couchman–Karasz equation to predict the T_g_ values, which may present a possible reason for the discrepancies in the results between the two studies. Although limited information has been reported on T–T_g_ values for WPC powders, the T_f_–T_g_ values obtained for the WPC 35 sample (23.1 °C and 19.9 °C for the midpoint and 15% RH, respectively) are in good agreement with T–T_g_ values reported for SMP of 20.6 °C and 23.3 °C by O’Donoghue et al. [18] and Hennigs et al. [8], respectively.

The T_α_–T_g_ results from DSC and the storage modulus of DMA (T_α_ onset), at both the midpoint and at 15% RH, show an overall decrease in T–T_g_ with increasing protein content for WPC powders, with the exception of the WPC 20. However, other studies have reported that the T–T_g_ of dairy powders increased with increasing protein content [16,36]. Nevertheless, for the lower protein powders, the T_α_–T_g_ values determined from the storage modulus are in good agreement with the T_f_–T_g_ results reported using the fluidization method in the current study (Table 4). However, for the higher protein powders, the T_α_–T_g_ values determined from the storage modulus are considerably lower than the T_f_–T_g_ fluidization results. Furthermore, as seen in Figure 6d, the α-relaxation curve generated from the storage modulus using DMA intersects the glass transition curve for the WPC 65 powder at an a_w_ of ~0.40. Therefore, in the current study, negative T–T_g_ values were observed for WPC 65 at a_w_ ≤ 0.40 (Table 4), i.e., the reported α-relaxation temperatures (T_α_ onset) occurred below the T_g_. Many studies [5,13,36] have shown that the stickiness curve typically tracks the glass transition curve for dairy powders, an observation that is also evident in the current study for the fluidization and glass transition curves of all four powders (Figure 6). However, in the case of the DMA (T_α_ onset) curve of the WPC 65 powder, the intersection with the glass transition curve is likely due to the fact that the DMA appears to also be measuring the same structural transition as the DSC.

In the study by Silalai and Roos [23], the authors compared the stickiness results to the α-relaxation results from the loss modulus (T_α_ peak). In the present study, the T_α_–T_g_ values calculated from the peaks of the loss moduli range from 20.6–27.9 °C and 17.7–28.4 °C for the four WPC powders at the midpoint and 15% RH, respectively. Maidannyk and Roos [17] reported similar T_α_–T_g_ results of ~20–30 °C for a variety of humidified WPI/lactose powders measured using DMA (T_α_ peak) and DSC. Similarly, Bengoechea et al. [44] reported T_α_–T_g_ values in the range of ~25–40 °C when comparing the T_α_ peak values from DMA to the T_g_ values measured by DSC for samples of casein and soy protein isolate (SPI). In the present study, the T_α_–T_g_ results obtained from the loss modulus (T_α_ peak) were consistently higher than the equivalent results for the storage modulus (T_α_ onset) (Table 4). While the T–T_g_ results for the lower protein powders were higher than those reported for the fluidization technique, the T–T_g_ values found for the loss modulus are more representative of the fluidization results for the higher protein powders. Overall, these T–T_g_ results suggest that for powders with protein contents less than approximately 45% *w*/*w*, the results obtained from the T_α_ onset values of the DMA method compare well to the T–T_g_ obtained from the fluidization technique and those reported in the literature. However, for higher protein powders, the T–T_g_ results determined from the T_α_ peak values of the DMA method may be more representative of the fluidization results.

## 4. Conclusions

DMA was shown to be an interesting complementary technique to other commonly applied methods for measurement of phase transitions (T_g_ by DSC) and stickiness behavior (fluidization technique) for dairy powders. The data demonstrated that the comparability to other techniques depends on the composition of the powder and the modulus used (T_α_ onset or T_α_ peak). The storage modulus results were in good agreement with the stickiness results from the fluidization technique for lower protein dairy powders (<45% protein *w*/*w*), whereas for powders with higher protein contents (~45–65% protein *w*/*w*), the results from the loss modulus were found to be more accurate. While DMA may not be a suitable method for stickiness determination, it has potential as a complementary technique that would provide more detailed information on the visco-elastic changes occurring during stickiness development. For example, the results of the current study suggest two different mechanisms of stickiness development: for the lower protein powders, stickiness occurs following a reduction in powder stiffness, and for the higher protein powder, there appears to be a two-stage mechanism involving a reduction in stiffness followed by a significant change in viscosity. It should also be noted that DMA is commonly used for T_g_ determination, and in the current study, the T_α_ onset results were found to be almost identical to the T_g_ results obtained using DSC analysis for the WPC 65.

Overall, this study has highlighted the variability of different methods reported in the literature to ostensibly measure the same or similar structural changes in dairy powders (i.e., SPT, T_g_) and has demonstrated that the use of material characterization methods, such as DMA, may facilitate a deeper understanding of the fundamental mechanisms of stickiness development. However, the static nature of the powders during DMA determination limits its applicability as a direct method to determine the stickiness of powders in a dynamic system such as a spray dryer. Therefore, it is recommended to use DMA in combination with methods such as fluidization or particle gun analyses which are more reflective of stickiness under dynamic conditions.

## Figures and Tables

**Table 1:** Composition of whey protein concentrate (WPC) powders with protein contents ranging from ~20 (WPC 20) to ~65% (WPC 65).

Sample	Protein (% *w*/*w*)	Fat (% *w*/*w*)	Lactose * (% *w*/*w*)	Ash (% *w*/*w*)	Free Moisture (% *w*/*w*) **	Particle Size D4,3 *** (µm)
WPC 20	19.3 ± 0.02 ^a^	1.31 ± 0.04 ^a^	66.2	7.34 ± 0.01 ^a^	5.90 ± 0.01 ^a^	106
WPC 35	35.7 ± 0.20 ^b^	3.19 ± 0.06 ^b^	48.6	6.36 ± 0.00 ^b^	6.11 ± 0.08 ^b^	118
WPC 50	53.4 ± 0.15 ^c^	4.15 ± 0.02 ^c^	32.8	5.59 ± 0.24 ^c^	4.07 ± 0.14 ^c^	118
WPC 65	69.1 ± 0.38 ^d^	5.33 ± 0.03 ^d^	17.5	4.23 ± 0.00 ^d^	3.90 ± 0.05 ^d^	115

* Calculated by difference; ** on a wet basis; *** D4,3 = volume mean diameter. ^a–d^ Within a column, values with different superscripts vary significantly (*p* < 0.05).

**Table 2:** Surface composition of whey protein concentrate (WPC) powders with protein contents ranging from ~20 (WPC 20) to ~65% (WPC 65).

Sample	Protein (%)	Lactose (%)	Fat (%)
WPC 20	46.8 ± 0.50 ^a^	47.8 ± 0.11 ^a^	4.07 ± 0.38 ^a^
WPC 35	48.9 ± 5.48 ^a,b^	33.8 ± 0.82 ^b^	16.7 ± 6.39 ^a,b^
WPC 50	52.5 ± 1.49 ^a,b^	23.5 ± 2.29 ^c^	23.9 ± 3.70 ^b^
WPC 65	59.2 ± 4.98 ^b^	13.6 ± 2.75 ^d^	27.2 ± 7.76 ^b^

^a–d^ Within a column, values with different superscripts vary significantly (*p* < 0.05).

**Table 3:** Onset temperatures for glass transition (T_g_) of whey protein concentrate (WPC) powders with protein contents ranging from ~20% (WPC 20) to ~65% (WPC 65), stored at different water activities (a_w_).

Sample	0.11 a_w_	0.23 a_w_	0.33 a_w_	0.44 a_w_
WPC 20	63.2 ± 0.56 ^a^	50.6 ± 0.00 ^a^	37.1 ± 0.25 ^a^	18.6 ± 0.07 ^a^
WPC 35	62.2 ± 0.01 ^b^	49.9 ± 0.17 ^b^	40.9 ± 0.60 ^b^	21.6 ± 0.01 ^b^
WPC 50	64.4 ± 0.13 ^c^	47.6 ± 0.30 ^c^	44.8 ± 0.26 ^c^	33.7 ± 0.42 ^c^
WPC 65	67.8 ± 0.23 ^d^	52.4 ± 0.10 ^d^	47.3 ± 0.23 ^d^	42.7 ± 0.03 ^d^

^a–d^ Within a column, values with different superscripts vary significantly (*p* < 0.05).

**Table 4:** Difference between sticky point (T_f_) or α-relaxation temperature (T_α_) and glass transition temperature (T_g_), determined for whey protein concentrate (WPC) powders with protein contents ranging from ~20% (WPC 20) to ~65% (WPC 65), at the midpoint of the stickiness curve and at 15% relative humidity (RH) using the fluidization or dynamic mechanical analysis (DMA) approach.

Method	Sample	T–Tg at Midpoint (°C)	T–Tg at 15% RH (°C)
Fluidization, T_f_	WPC 20	18.1	13.2
	WPC 35	23.1	19.9
	WPC 50	22.7	29.5
	WPC 65	18.0	28.4
DMA, T_α_ onset	WPC 20	18.3	18.1
	WPC 35	21.5	21.1
	WPC 50	15.1	19.5
	WPC 65	−1.14	−2.35
DMA, T_α_ peak	WPC 20	26.6	28.1
	WPC 35	27.9	28.4
	WPC 50	27.1	22.7
	WPC 65	20.6	17.7

## References

[1] Chuy L.E., Labuza T.P. (1994). Caking and stickiness of dairy-based food powders as related to glass transition. J. Food Sci..

[2] Paterson A.H.J., Bronlund J.E., Brooks G.F. The blow test for measuring the stickiness of powders. Proceedings of the AIChE 2001 Annual Meeting.

[3] Özkan N., Walisinghe N., Chen X.D. (2002). Characterization of stickiness and cake formation in whole and skim milk powders. J. Food Eng..

[4] Zuo J.Y., Paterson A.H.J., Bronlund J.E., Chatterjee R. (2007). Using a particle-gun to measure initiation of stickiness of dairy powders. Int. Dairy J..

[5] Hogan S., O’Callaghan D., Bloore G. (2009). Application of fluidised bed stickiness apparatus to dairy powder production. Milchwissenschaft.

[6] Silalai N., Roos Y.H. (2011). Mechanical relaxation times as indicators of stickiness in skim milk–maltodextrin solids systems. J. Food Eng..

[7] Lazar M., Brown A., Smith G., Wong F., Lindquist F. (1956). Experimental production of tomato powder by spray drying. Food Technol..

[8] Hennigs C., Kockel T., Langrish T. (2001). New measurements of the sticky behavior of skim milk powder. Dry. Technol..

[9] Murti R.A., Paterson A.H.J., Pearce D.L., Bronlund J.E. (2010). The influence of particle velocity on the stickiness of milk powder. Int. Dairy J..

[10] Brooks G.F. (2000). The Sticking and Crystallisation of Amorphous Lactose. Master’s Thesis.

[11] Couchman P., Karasz F. (1978). A classical thermodynamic discussion of the effect of composition on glass-transition temperatures. Macromolecules.

[12] Jouppila K., Roos Y. (1994). Glass transitions and crystallization in milk powders. J. Dairy Sci..

[13] Ozmen L., Langrish T. (2002). Comparison of glass transition temperature and sticky point temperature for skim milk powder. Dry. Technol..

[14] Haque M.K., Roos Y. (2004). Water plasticization and crystallization of lactose in spray-dried lactose/protein mixtures. J. Food Sci..

[15] Haque M.K., Roos Y. (2004). Water sorption and plasticization behavior of spray-dried lactose/protein mixtures. J. Food Sci..

[16] Silalai N., Roos Y.H. (2010). Roles of water and solids composition in the control of glass transition and stickiness of milk powders. J. Food Sci..

[17] Maidannyk V., Roos Y. (2017). Water sorption, glass transition and “strength” of lactose-whey protein systems. Food Hydrocoll..

[18] O’Donoghue L.T., Haque M.K., Kennedy D., Laffir F.R., Hogan S.A., O’Mahony J.A., Murphy E.G. (2019). Influence of particle size on the physicochemical properties and stickiness of dairy powders. Int. Dairy J..

[19] Roos Y., Karel M. (1991). Plasticizing effect of water on thermal behavior and crystallization of amorphous food models. J. Food Sci..

[20] Karel M., Anglea S., Buera P., Karmas R., Levi G., Roos Y. (1994). Stability-related transitions of amorphous foods. Thermochim. Acta.

[21] Boonyai P., Bhandari B., Howes T. (2014). Stickiness measurement techniques for food powders: A review. Powder Technol..

[22] Murti R.A., Paterson A.H.J., Pearce D.L., Bronlund J.E. (2009). Stickiness of skim milk powder using the particle gun technique. Int. Dairy J..

[23] Silalai N., Roos Y.H. (2011). Coupling of dielectric and mechanical relaxations with glass transition and stickiness of milk solids. J. Food Eng..

[24] Fan F., Roos Y.H. (2016). Structural relaxations of amorphous lactose and lactose-whey protein mixtures. J. Food Eng..

[25] Fan F., Roos Y.H. (2017). Structural strength and crystallization of amorphous lactose in food model solids at various water activities. Inn. Food Sci. Emerg. Technol..

[26] IDF (1987). Determination of Fat Content—Rose Gottlieb Reference Method. IDF Standard 9C.

[27] Faldt P., Bergenstahl B., Carlsson G. (1993). The surface coverage of fat on food powders analyzed by ESCA (electron spectroscopy for chemical analysis). Food Struct..

[28] Murphy E.G., Roos Y.H., Hogan S.A., Maher P.G., Flynn C.G., Fenelon M.A. (2015). Physical stability of infant milk formula made with selectively hydrolysed whey proteins. Int. Dairy J..

[29] Nijdam J., Langrish T. (2006). The effect of surface composition on the functional properties of milk powders. J. Food Eng..

[30] Shrestha A.K., Howes T., Adhikari B.P., Wood B.J., Bhandari B.R. (2007). Effect of protein concentration on the surface composition, water sorption and glass transition temperature of spray-dried skim milk powders. Food Chem..

[31] Kim E.H.-J., Chen X.D., Pearce D. (2009). Surface composition of industrial spray-dried milk powders. 2. Effects of spray drying conditions on the surface composition. J. Food Eng..

[32] Foerster M., Gengenbach T., Woo M.W., Selomulya C. (2016). The impact of atomization on the surface composition of spray-dried milk droplets. Colloids Surf. B Biointerfaces.

[33] Maidannyk V., McSweeney D.J., Hogan S.A., Miao S., Montgomery S., Auty M.A., McCarthy N.A. (2020). Water sorption and hydration in spray-dried milk protein powders: Selected physicochemical properties. Food Chem..

[34] Berlin E., Anderson B.A., Pallansch M.J. (1968). Comparison of water vapor sorption by milk powder components. J. Dairy Sci..

[35] Foster K.D., Bronlund J.E., Paterson A.H.J. (2005). The prediction of moisture sorption isotherms for dairy powders. Int. Dairy J..

[36] Hogan S., O’Callaghan D. (2010). Influence of milk proteins on the development of lactose-induced stickiness in dairy powders. Int. Dairy J..

[37] Downton G.E., Flores-Luna J.L., King C.J. (1982). Mechanism of stickiness in hygroscopic, amorphous powders. Ind. Eng. Chem. Fund..

[38] Menard K.P. (2002). Dynamic Mechanical Analysis. Encyclopedia of Polymer Science and Technology.

[39] Kalichevsky M., Jaroszkiewicz E., Ablett S., Blanshard J., Lillford P. (1992). The glass transition of amylopectin measured by DSC, DMTA and NMR. Carbohydr. Polym..

[40] Rahman M.S., Al-Marhubi I.M., Al-Mahrouqi A. (2007). Measurement of glass transition temperature by mechanical (DMTA), thermal (DSC and MDSC), water diffusion and density methods: A comparison study. Chem. Phys. Lett..

[41] Kararli T.T., Hurlbut J.B., Needham T.E. (1990). Glass-rubber transitions of cellulosic polymers by dynamic mechanical analysis. J. Pharm. Sci..

[42] Hallberg L., Chinachoti P. (1992). Dynamic mechanical analysis for glass transitions in long shelf-life bread. J. Food Sci..

[43] Siebenmorgen T., Yang W., Sun Z. (2004). Glass transition temperature of rice kernels determined by dynamic mechanical thermal analysis. Trans. ASAE.

[44] Bengoechea C., Arrachid A., Guerrero A., Hill S.E., Mitchell J.R. (2007). Relationship between the glass transition temperature and the melt flow behavior for gluten, casein and soya. J. Cereal Sci..

[45] Schuck P., Dolivet A., Méjean S., Jeantet R. (2008). Relative humidity of outlet air: The key parameter to optimize moisture content and water activity of dairy powders. Dairy Sci. Technol..

